# Analysis of the departmental availability of nutritionists and indicators of anemia and maternal-child malnutrition in Peru

**DOI:** 10.17843/rpmesp.2026.431.15527

**Published:** 2026-02-21

**Authors:** Jamee Guerra Valencia, Akram Hernández-Vásquez

**Affiliations:** 1 Facultad de Ciencias de la Salud, Universidad Privada del Norte, Lima, Peru.; 2 Universidad San Ignacio de Loyola, Vicerrectorado de Investigación, Centro de Excelencia en Investigaciones Económicas y Sociales en Salud, Lima, Peru.

To the editor. Historically, maternal and child malnutrition in Peru has represented a significant health and economic burden ^1^. Although improvements have been documented in some nutritional indicators, high levels of anemia and maternal and child malnutrition persist ^1^, highlighting the persistence of preventable conditions that affect critical stages of life.

In this context, nutritionists play an essential role in the prevention and reduction of nutritional problems. In Peru, Law 30188 recognizes nutritionists as a health professionals, and Technical Health Standard No. 103 (NTS 103) for nutrition and dietetic services ^2^ establishes specific parameters for hospital care, such as requiring one nutritionist per 40 hospital beds or per 15 patients in intensive care units. However, standards have not been defined for the general population or for primary health care settings, where most healthcare services are delivered. This lack of staffing standards not only contributes to the persistence of nutritional problems but also limits the achievement of national and international malnutrition reduction targets . Therefore, the objective of this letter was to describe the availability and distribution of nutritionists in the public health system during 2024.

A descriptive analysis of official sources was conducted. Data from the Registro Nacional del Personal de la Salud (INFORHUS) provided the number of nutritionists registered in the public health system, which was expressed as the number of nutritionists per 100,000 inhabitants at the department level. Prevalence estimates of anemia and stunting in children under five, as well as anemia and underweight in pregnant women, were obtained from the Tableros de Indicadores del Centro Nacional de Alimentación, Nutrición y Vida Saludable (SIEN) at the department level. Using these data, quadrant plots and correlation analyses were performed, where each point represents a department and its position in the plot reflects the relationship between the nutritionist rate and the magnitude of nutritional problems, including reference lines at the medians and a trend line.

**Figure 1:**
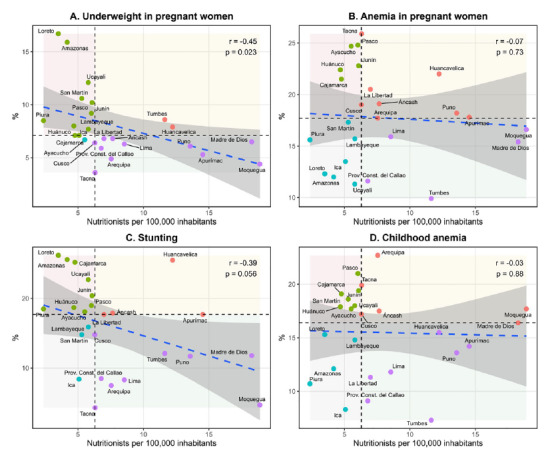
Correlation and quadrants between the rate of nutritionists per 100,000 inhabitants and the percentage of nutritional problems at the departmental level in Peru in 2024. A) Underweight in pregnant women, B) Anemia in pregnant women, C) Stunting, and D) Childhood anemia.

The rate of nutritionists in the public health system showed marked variability across departments, with approximately an eightfold difference between the lowest value (Piura) and the highest (Moquegua) (2.4 vs. 18.9 per 100,000 inhabitants). The quadrant analysis identified departments with both a high prevalence of nutritional problems and a low rate of nutritionists, such as Pasco, Junín, and Huánuco. Correlation analysis revealed a negative relationship between the rate of nutritionists and underweight in pregnant women, as well as stunting in children. No correlation was observed with anemia in either children or pregnant women (Figure 1).

Data on nutritionists were obtained from the Registro Nacional del Personal de la Salud, with a cutoff date of December 2024 (https://files.minsa.gob.pe/s/8HxQA-JT8Ez8b27F/download/base%20diciembre_2024-REPORTE%20pag%20web%20MINSA%20Y%20GORES.xlsx), while the departmental population for 2024 corresponds to the estimated population as of June 30, by calendar year and sex, according to department, 2000-2025 series published by the INEI (https://m.inei.gob.pe/estadisticas/indice-tematico/population-estimates-and-projections/). Nutritional status indicators for 2024 were obtained from the Tableros de Indicadores del Centro Nacional de Alimentación, Nutrición y Vida Saludable, specifically: underweight in pregnant women (https://www.minsa.gob.pe/reunis/data/sien-hisminsa-es-tado-nuticional-gestantes.asp), stunting in children under five (https://www.minsa.gob.pe/reunis/data/sien-hisminsa-5.asp), and anemia in pregnant women and in children under 5 years of age (https://www.minsa.gob.pe/reunis/data/sien-hisminsa-anemia-5.asp). For the department of Lima, data from the Direcciones de Redes Integradas de Salud (DIRIS) were aggregated into a single category. Quadrants were defined based on the medians of the nutritionist rate (median = 6.29) and each nutritional indicator (underweight in pregnant women: median = 7.1; anemia in pregnant women: median = 17.7; stunting: median = 17.7; childhood anemia: median = 16.4), where each quadrant represents combinations of high/low nutritionist rates and high/low prevalence of nutritional problems. Pearson correlations coefficients (r) and their corresponding p-values (p) are presented.

These findings demonstrate substantial variability in the distribution of nutritionists across departments. NTS 103 represented an advance by regulating hospital settings through bed-based ratios; however, gaps remain in primary health care and community care settings. The World Health Organization has noted that the Americas have the highest rate of nutritionists but also the greatest variability among countries ^3^. This inequality is also observed within Peru, where the rate of nutritionists varies nearly eightfold between departments and, in regions such as Junín, Pasco, and Huánuco, a high prevalence of malnutrition indicators coexists with a low rate of nutritionists, reflecting limitations in health system planning. In contrast, in Spain, a ratio of one dietitian-nutritionist per 50,000 health cards in primary care has been proposed, along with defined hospital ratios ^4^.

The need to close this gap is further underscored by the Multisectoral Plan for the Prevention and Reduction of Maternal and Child Anemia 2024-2030, which allocated more than PEN 542 million to activities such as nutritional care for children with anemia, promotion and education on complementary feeding, and education on the consumption of fortified foods ^5^, all of which require the participation of nutritionists. The magnitude of this funding contrasts with the availability of nutritionists in 2024, when 1,490 SERUMS positions were offered, representing 5% of the total number SERUMS positions (see Supplementary material), which may limit the effectiveness of interventions and compromise the expected impact. Furthermore, the redistribution of human resources alone may be insufficient unless accompanied by strategies for capacity building, standardization of interventions, and continuous training in nutrition education and comprehensive management of nutritional problems.

In conclusion, the results highlight the absence of staffing standards for nutritionists at the primary care level, precisely where the burden of maternal and child malnutrition is greatest. This structural gap in health system planning limits the extent to which of nutrition-related investments translates into effective public health improvements. Effective staffing of nutritionists at these levels would help reduce territorial inequalities and enhance the impact of such investments.
