# Supplementary material for: Analysis of the departmental availability of nutritionists and indicators of anemia and maternal-child malnutrition in Peru
Source: Rev Peru Med Exp Salud Publica. 2026 Feb 21;43(1):143–5. doi: 10.17843/rpmesp.2026.431.15527 (PMC13245977; doi:10.17843/rpmesp.2026.431.15527)
Supplement: Supplementary material. — Available in the electronic version of the RPMESP. [file rpmesp-43-01-15527-s001.docx]

**MATERIAL SUPLEMENTARIO**

| **Tabla S1.** Plazas de SERUMS para las diferentes profesiones durante el año 2024 (n = 29 704) | |
| --- | --- |
| **Profesión** | **n (%)** |
| Biología | 968 (3,3) |
| Enfermería | 5052 (17,0) |
| Ingeniería sanitaria | 74 (0,2) |
| Medicina | 5005 (16,8) |
| Medicina con especialidad | 22 (0,1) |
| Medicina Veterinaria | 265 (0,9) |
| Nutrición | 1490 (5,0) |
| Obstetricia | 2845 (9,6) |
| Odontología | 2108 (7,1) |
| Psicología | 7485 (25,2) |
| Químico Farmacéutico | 1445 (4,9) |
| Tecnología Médica - Laboratorio Clínico | 1054 (3,5) |
| Tecnología Médica - Optometría | 71 (0,2) |
| Tecnología Médica - Radiología | 297 (1,0) |
| Tecnología Médica - Terapia Física | 532 (1,8) |
| Tecnología Médica - Terapia de Lenguaje | 131 (0,4) |
| Tecnología Médica - Terapia Ocupacional | 108 (0,4) |
| Trabajo Social | 752 (2,5) |

Incluye ambas modalidades de plazas de SERUMS (remunerada y equivalente) de los procesos 2024-I y 2024-II.

La información se obtuvo a partir de los datos disponibles en:

1. Proceso 2024-I: https://www.gob.pe/institucion/minsa/informes-publicaciones/5284567-oferta-de-plazas-serums-remuneradas-y-equivalentes
2. Proceso 2024-II: https://www.gob.pe/institucion/minsa/informes-publicaciones/5970113-oferta-de-plazas-serums-remuneradas-y-equivalentes

| **Tabla S2.** Plazas de SERUMS para profesionales de Nutrición por departamento durante el año 2024 (n = 1490) | |
| --- | --- |
| **Departamento** | **n (%)** |
| Amazonas | 12 (0,8) |
| Ancash | 44 (3,0) |
| Apurímac | 25 (1,7) |
| Arequipa | 84 (5,6) |
| Ayacucho | 47 (3,2) |
| Cajamarca | 36 (2,4) |
| Callao | 81 (5,4) |
| Cusco | 78 (5,2) |
| Huancavelica | 29 (1,9) |
| Huánuco | 11 (0,7) |
| Ica | 43 (2,9) |
| Junín | 54 (3,6) |
| La Libertad | 60 (4,0) |
| Lambayeque | 63 (4,2) |
| Lima | 521 (35,0) |
| Loreto | 20 (1,3) |
| Madre De Dios | 9 (0,6) |
| Moquegua | 21 (1,4) |
| Pasco | 12 (0,8) |
| Piura | 29 (1,9) |
| Puno | 109 (7,3) |
| San Martín | 7 (0,5) |
| Tacna | 40 (2,7) |
| Tumbes | 25 (1,7) |
| Ucayali | 30 (2,0) |

Incluye ambas modalidades de plazas de SERUMS (remunerada y equivalente) de los procesos 2024-I y 2024-II.

La información se obtuvo a partir de los datos disponibles en:

1. Proceso 2024-I: https://www.gob.pe/institucion/minsa/informes-publicaciones/5284567-oferta-de-plazas-serums-remuneradas-y-equivalentes
2. Proceso 2024-II: https://www.gob.pe/institucion/minsa/informes-publicaciones/5970113-oferta-de-plazas-serums-remuneradas-y-equivalentes
