# Supplementary material for: Analysis of the departmental availability of nutritionists and indicators of anemia and maternal-child malnutrition in Peru
Source: Rev Peru Med Exp Salud Publica. 2026 Feb 21;43(1):143–5. doi: 10.17843/rpmesp.2026.431.15527 (PMC13245977; doi:10.17843/rpmesp.2026.431.15527)
Supplement: Translation

# Translation: Análisis de la disponibilidad departamental de nutricionistas e indicadores de anemia y malnutrición materno-infantil en el Perú

**Authors:** Jamee Guerra Valencia, Akram Hernández-Vásquez

**Affiliations:** 1 Facultad de Ciencias de la Salud, Universidad Privada del Norte, Lima, Perú.; 2 Universidad San Ignacio de Loyola, Vicerrectorado de Investigación, Centro de Excelencia en Investigaciones Económicas y Sociales en Salud, Lima, Perú.

Sr. Editor. Históricamente, la malnutrición materno-infantil ha representado en el Perú una importante carga de enfermedad y económica ^1^. Aunque se han documentado mejoras en algunos indicadores nutricionales, persisten niveles elevados de anemia y desnutrición materno-infantil ^1^, evidenciando la continuidad de problemas prevenibles que afectan etapas críticas de la vida.

En este contexto, los nutricionistas cumplen un rol esencial para la prevención y reducción de los problemas nutricionales. En el Perú, la Ley 30188 reconoce al nutricionista como profesional de la salud, y la Norma Técnica de Salud N.° 103 (NTS 103) sobre servicios de nutrición y dietética ^2^, establece algunos parámetros para la atención hospitalaria, como contar con un nutricionista por cada 40 camas hospitalarias o cada 15 pacientes en cuidados intensivos. Sin embargo, no se han definido estándares para la población general ni para la atención primaria, donde se concentra la mayor parte de la atención sanitaria. Esta ausencia de criterios de dotación no solo favorece la persistencia de los problemas nutricionales, sino que también limita el cumplimiento de las metas nacionales e internacionales de reducción de la malnutrición. Por ello, el objetivo de la presente carta fue describir la disponibilidad y distribución de nutricionistas en el sistema público de salud durante el año 2024.

Se realizó un análisis descriptivo de fuentes oficiales. La base de datos del Registro Nacional del Personal de la Salud (INFORHUS) proporcionó el número de nutricionistas registrados en el sistema público de salud, que se estandarizó como tasa de nutricionistas por cada 100 000 habitantes a nivel departamental. De los tableros de indicadores del Sistema de Información del Estado Nutricional (SIEN) se obtuvieron las prevalencias de anemia y desnutrición crónica en menores de cinco años, así como de anemia y déficit de peso en gestantes a nivel departamental. Con estos datos se elaboraron gráficos de cuadrantes y correlación donde cada punto representa un departamento y su relación con la tasa de nutricionistas y la magnitud de problemas nutricionales, incluyendo líneas de referencia en las medianas y recta de tendencia.

La tasa de nutricionistas en el sistema público de salud mostró una marcada variabilidad entre departamentos, con diferencias de alrededor ocho veces entre el valor más bajo (Piura) y el más alto (Moquegua) (2,4 vs. 18,9 por 100 000 habitantes). El análisis de cuadrantes encontró departamentos con alta prevalencia de problemas nutricionales y baja tasa de nutricionistas, como Pasco, Junín y Huánuco. El análisis de correlación identificó una relación negativa entre la tasa de nutricionistas y el déficit de peso en gestantes, así como también para la desnutrición crónica infantil. No se observó correlación con la anemia infantil ni en gestantes (Figura 1).

**Figura 1:** Correlación y cuadrantes entre la tasa de nutricionistas por 100 000 habitantes y porcentaje de problemas nutricionales a nivel departamental en el Perú en el 2024. A) déficit de peso en gestantes, B) anemia en gestantes, C) desnutrición crónica infantil y D) anemia infantil.

Los datos de nutricionistas se obtuvieron del Registro Nacional del Personal de la Salud con fecha de corte a diciembre de 2024 (https://files.minsa.gob.pe/s/8HxQAJT8Ez8b27F/download/base%20diciembre_2024-REPORTE%20pag%20web%20MINSA%20Y%20GORES.xlsx), mientras que la población departamental del año 2024 corresponde a la serie de Población estimada al 30 de junio, por años calendario y sexo, según departamento, 2000-2025, publicadas por el INEI (https://m.inei.gob.pe/estadisticas/indice-tematico/population-estimates-and-projections/). Los indicadores de estado nutricional para el 2024 provienen de los Tableros de Indicadores del Centro Nacional de Alimentación, Nutrición y Vida Saludable, específicamente: déficit de peso en gestantes (https://www.minsa.gob.pe/reunis/data/sien-hisminsa-estado-nuticional-gestantes.asp), desnutrición crónica infantil https://www.minsa.gob.pe/reunis/data/sien-hisminsa-5.asp) y anemia en gestantes y en menores de 5 años (https://www.minsa.gob.pe/reunis/data/sien-hisminsa-anemia-5.asp). Para el departamento de Lima, los datos de las Direcciones de Redes Integradas de Salud (DIRIS) se agruparon en una sola categoría. Los cuadrantes se definieron según las medianas de la tasa de nutricionistas (mediana = 6,29) y de cada indicador nutricional (déficit de peso en gestantes: mediana = 7,1; anemia en gestantes: mediana = 17,7; desnutrición crónica infantil = 17,7; anemia infantil: mediana = 16,4), donde cada cuadrante representa combinaciones de alta/baja tasa de nutricionistas y alta/baja prevalencia de problemas nutricionales. Se presentan las correlaciones de Pearson (r) y sus valores de p (p).

Los hallazgos evidencian una marcada variabilidad en la tasa de nutricionistas entre departamentos. La NTS 103 representó un avance al regular el ámbito hospitalario con ratios por camas, pero se mantienen vacíos en la atención primaria y comunitaria. A nivel internacional, la Organización Mundial de la Salud ha señalado que las Américas presenta la mayor tasa de nutricionistas, pero también la mayor dispersión entre países ^3^. Esta desigualdad se replica dentro del Perú, donde la tasa de nutricionistas difiere casi ocho veces entre departamentos y, en regiones como Junín, Pasco y Huánuco, coexiste alta prevalencia de los indicadores de malnutrición evaluados con baja tasa de nutricionistas, lo que refleja limitaciones en la planificación sanitaria. En contraste, en España se ha propuesto un dietista-nutricionista por cada 50 000 tarjetas sanitarias en atención primaria y ratios definidos en hospitales ^4^.

La necesidad de cerrar esta brecha se refuerza al considerar que el Plan Multisectorial para la Prevención y Reducción de la Anemia Materno Infantil 2024-2030 asignó más de S/ 542 millones a actividades de atención nutricional de niñas y niños con anemia, promoción y educación en el uso de alimentos complementarios y a la educación sobre el consumo de alimentos fortificados ^5^, cuya ejecución involucra la participación de nutricionistas. La magnitud de este financiamiento contrasta con la disponibilidad de nutricionistas durante el 2024, año en el que se ofertaron 1490 plazas SERUMS, equivalentes al 5% del total de plazas SERUMS (Ver Material Suplementario), lo que podría limitar la efectividad de las intervenciones y comprometer el impacto esperado. Asimismo, la redistribución del recurso humano por sí sola puede resultar insuficiente si no se acompaña de estrategias de fortalecimiento de capacidades, estandarización de intervenciones y formación continua en educación alimentaria y manejo integral de problemas nutricionales.

En conclusión, los resultados resaltan la ausencia de estándares de dotación de nutricionistas en el primer nivel de atención, precisamente en los ámbitos donde se concentra la mayor carga de malnutrición materno-infantil. Esta brecha estructural en la planificación sanitaria limita que los recursos destinados a nutrición se traduzcan en mejoras efectivas de la salud pública. Una dotación efectiva de nutricionistas en estos niveles permitiría reducir desigualdades territoriales y potenciar el impacto de dichas inversiones.
